# Alanine Enhances Aminoglycosides-Induced ROS Production as Revealed by Proteomic Analysis

**DOI:** 10.3389/fmicb.2018.00029

**Published:** 2018-01-30

**Authors:** Jin-zhou Ye, Yu-bin Su, Xiang-min Lin, Shi-shi Lai, Wan-xin Li, Farman Ali, Jun Zheng, Bo Peng

**Affiliations:** ^1^Center for Proteomics and Metabolomics, State Key Laboratory of Bio-Control, School of Life Sciences, Sun Yat-sen University, Guangzhou, China; ^2^Laboratory for Marine Biology and Biotechnology, Qingdao National Laboratory for Marine Science and Technology, Qingdao, China; ^3^Fujian Provincial Key Laboratory of Agroecological Processing and Safety Monitoring, Key Laboratory of Crop Ecology and Molecular Physiology, College of Life Sciences, Fujian Agriculture and Forestry University, Fuzhou, China; ^4^Faculty of Health Sciences, University of Macau, Macau, China

**Keywords:** alanine, proteomics, metabolomics, reactive oxygen species, riboflavin metabolism

## Abstract

Metabolite-enabled killing of antibiotic-resistant pathogens by antibiotics is an attractive strategy to manage antibiotic resistance. Our previous study demonstrated that alanine or/and glucose increased the killing efficacy of kanamycin on antibiotic-resistant bacteria, whose action is through up-regulating TCA cycle, increasing proton motive force and enhancing antibiotic uptake. Despite the fact that alanine altered several metabolic pathways, other mechanisms could be potentially involved in alanine-mediated kanamycin killing of bacteria which remains to be explored. In the present study, we adopted proteomic approach to analyze the proteome changes induced by exogenous alanine. Our results revealed that the expression of three outer membrane proteins was altered and the deletion of *nagE* and *fadL* decreased the intracellular kanamycin concentration, implying their possible roles in mediating kanamycin transport. More importantly, the integrated analysis of proteomic and metabolomic data pointed out that alanine metabolism could connect to riboflavin metabolism that provides the source for reactive oxygen species (ROS) production. Functional studies confirmed that alanine treatment together with kanamycin could promote ROS production that in turn potentiates the killing of antibiotic-resistant bacteria. Further investigation showed that alanine repressed the transcription of antioxidant-encoding genes, and alanine metabolism to riboflavin metabolism connected with riboflavin metabolism through TCA cycle, glucogenesis pathway and pentose phosphate pathway. Our results suggest a novel mechanism by which alanine facilitates kanamycin killing of antibiotic-resistant bacteria via promoting ROS production.

## Introduction

The widespread of antibiotic-resistant bacteria is a growing problem, imposing catastrophic threat to people in every country throughout the world. The control of antibiotic-resistant bacteria becomes an urgent issue to the society. Although governmental interventions are undertaken to control the use of antibiotics in clinics and poultry industry, the approach to eliminate the existing antibiotic-resistant bacteria is still limited. Developing novel classes of antibiotics as well as antibiotic derivatives through chemical modifications to provide antibiotic derivatives are the main strategies by pharmaceutical companies and health care systems to eliminate resistance (Bush et al., 2011). However, the approach is difficult given the fact that the industry’s search of novel chemical agents acting on new biological targets is proven to be non-productive (Payne et al., 2007; Laxminarayan et al., 2013; Ardal et al., 2017). Another major strategy is the non-antibiotic approach including antibacterial vaccines, phage therapy, immunostimulants, adjuvants, anti-virulence therapies, probiotics and their combinations (Alekshun and Levy, 2004; Bush et al., 2011). Unfortunately, the development of non-antibiotic approaches lagged behind the expectation, and meet limited success (Coates and Hu, 2007; Raju et al., 2012).

The major challenge to kill antibiotic-resistant bacteria is the limited concentration of antibiotics that can be achieved inside bacterial cells, which is likely due to the elevated efflux or reduced influx of the antibiotics (Pagès et al., 2008). Novel approaches thus are required to overcome this limitation to increase the intracellular antibiotic concentration to a certain threshold so that the resistant bugs can be killed. However, several lines of evidences demonstrated that microbial environment confounds antibiotic efficacy through metabolic processes (Lee and Collins, 2012). Metabolites like indole, produced by a subpopulation of bacteria but shared by all enabled the whole population to defend against antibiotic stress (Lee et al., 2010). Gas is another type of cytoprotective agent that protects bacteria against a wide range of antibiotics, e.g., nitric oxide alleviates antibiotic-induced ROS in bacteria thus prevent cell death (Gusarov et al., 2009). The micro-environment of bacteria community is thus determining antibiotic susceptibility, which provides the basis to engineer bacterial metabolic pathways to combat antibiotic resistance. Metabolites have been proved to be a useful way. The treatment of persisters, the highly antibiotic-tolerant subpopulation of the bacteria, with glucose, mannitol or fructose would greatly enhance the killing of persisters by aminoglycosides (Allison et al., 2011). Moreover, several recent studies highlight the importance of TCA cycle in fighting against multidrug resistant bacteria. The promotion of tricarboxylic cycle (TCA cycle) through exogenous alanine, glucose and fructose could greatly enhance the killing efficacy of kanamycin on different types of multidrug-resistant bacteria like *Vibrio parahaemolyticus*, *Klebsiella pneumoniae*, *Pseudomonas aeruginosa* and *Staphylococcus aureus*, persisters, and *in vivo* biofilm infections (Allison et al., 2011; Peng et al., 2015a; Su et al., 2015). The underlying mechanism involves the metabolites in promoting TCA cycle, increasing the generation of NADH, the substrates for proton motive force (PMF) production. The increased PMF ultimately increased intracellular concentration of kanamycin through enhanced antibiotic uptake. Thus, these studies highlighted the role of activation of TCA cycle in killing antibiotic-resistant bacteria by aminoglycosides (Peng et al., 2015b; Su et al., 2015). A later study further demonstrated that the tuning of TCA cycle could influence the antibiotic susceptibility of *Pseudomonas aeroginosa* to antibiotics (Meylan et al., 2017). Thus, the combinatorial use of metabolite and antibiotics has promising potential in eliminating the antibiotic-resistant bacteria by “reusing” old antibiotics.

The metabolic mechanism of alanine, glucose and fructose in potentiating kanamycin to kill antibiotic-resistant bacteria is well elucidated in our previous studies (Peng et al., 2015b; Su et al., 2015). However, whether other mechanisms have been involved in alanine and antibiotic-triggered cell death is still unexplored. In this study, we adopted proteomic approach to investigate the global proteome change in response to exogenous alanine. We found that exogenous alanine affects the expression of three outer membrane proteins. Furthermore, the integrated analysis of proteomic and metabolomics data directs our attention to ROS that can be synergistically produced by the combination of alanine and kanamycin. This study thus gains new insights on mechanisms of alanine-enabled killing of antibiotic-resistant bacteria by kanamycin.

## Results

### Proteomic Analysis of Alanine-Treated Antibiotic-Resistant Bacteria

In our previous report, we found that exogenous alanine reprogrammed the metabolome of *Edwardsiella tarda* EIB202, featured with twelve altered metabolic pathways (Peng et al., 2015b). Although the metabolomic data provided profound insights into how alanine modulates the metabolome of target cell and causes the death of multidrug-resistant bacteria by kanamycin, other biological processes that are involved might be neglected during metabolomics analysis. Thus, we implemented proteomic approach to further investigate the proteome change that is associated with exogenous alanine.

We continued using the wild-type multidrug-resistant *E. tarda* strain EIB202, and treated EIB202 with the dose of alanine (40 mM) we previously adopted (Peng et al., 2015b). After treatment, the whole cells were lysed, and total proteins were purified, labeled with iTRAQ and analyzed with LC-MS/MS. A total of 1972 protein were identified, where 40 proteins were differentially expressed as compared to the control group treated with saline buffer (fold of average change larger than 1.5 and *p* < 0.05 in both biological replicates is considered as differentially expressed proteins) (**Supplementary Table 1**). Among the differential proteins, the expression levels of 22 proteins were increased while 19 proteins were decreased (**Tables 1**, **2**).

**Table 1:** Lists of up-regulated proteins in *E. tarda* treated with alanine.

Name	Annotation	Peptides(95%)	%Coverage(95)	Folds of change
*asnA*	Aspartate–ammonia ligase	6	20	12.32
*ompA*	Outer membrane protein A	179	83.19	8.66
*sucC*	Succinate–CoA ligase [ADP-forming] subunit beta	63	69.07	7.02
*trpE*	Anthranilate synthase component 1	15	28.63	6.12
*ispG*	4-hydroxy-3-methylbut-2-en-1 -yl diphosphate synthasi	24	58.56	4.2
*esaJ*	EsaJ	20	45.68	4.18
*fadL*	Long-chain fatty acid transport protein	19	32.65	4.014
*cydA*	Cytochrome d terminal oxidase_:_ polypeptide subunit I	40	20.77	3.5
*ilvD*	Dihydroxy-acid dehydratase	25	40.58	3.42
*msrP*	Protein-methionine-sulfoxide reductase MsrP	17	47.45	3.11
*sucD*	Succinate—CoA ligase [ADP-forming] subunit alpha	30	48.62	2.73
*nagE*	PTS system. *N*-acetylglucosamine-specific IIBC subun	29	28.78	2.64
*ETAEJ421*	Predicted lipoprotein	12	42.27	2.49
*ilvA*	L-threonine dehydratase	12	26.85	2.2
*nifS*	Cysteine desulfurase IscS	68	64.85	2.14
*asnB*	Asparagine synthetase B	24	34.36	2.11
*ETAE_2952*	Uncharacterized protein	41	58.58	2
*hflC*	Protein HflC	27	59.58	1.97
*esaN*	EsaN	14	27.85	1.67
*ppiD*	Peptidylprolyl isomerase	50	51.6	1.47
*acrA*	Efflux transporter. RXD family. MFP subunit	47	60.15	1.45


**Table 2:** Lists of down-regulated proteins in *E. tarda* treated with alanine.

Name	Annotation	Peptides(95%)	%Coverage(95)	Folds of change
*ETAE_2682*	Alpha-1.4 glucan phosphorylase	44	39.53	0.55
*glpK*	Glycerol kinase	36	52.99	0.49
*adAl*	Lysine decarboxylase 1	289	66.2	0.44
*yeaG*	Putative Ser protein kinase	130	56.83	0.42
*ETAE_3167*	Uncharacterized protein	25	70.27	0.39
*clpA*	ATP-dependent Clp protease ATP-binding subunit	38	38.48	0.39
*ygeW*	Uncharacterized protein	22	46.58	0.34
*ETAE_3168*	Putative chlorohydrolase arainohydrolase	24	37.1	0.34
*evpB*	EvpB	42	63.03	0.31
*ETAE_0474*	Uncharacterized protein	7	27.97	0.31
*ETAE_1478*	UPF0229 protein ETAE1478	19	40.9	0.3
*evpK*	Type VI secretion system protein EvpK	38	61.13	0.3
*ETAE_2022*	Zinc metalloproteinase aureolysin	93	60.44	0.29
*evpG*	EvpG	8	23.75	0.29
*evpF*	EvpF	19	25.45	0.26
*evpH*	Type VI secretion system protein EvpH	94	71.95	0.18
*1540*	Amino-acid ABC transporter periplasmic component	65	82.88	0.16
*evpD*	Type VI secretion system protein EvpD	15	20	0.11
*evpC*	EvpC	75	74.85	0.08


**FIGURE 1:**
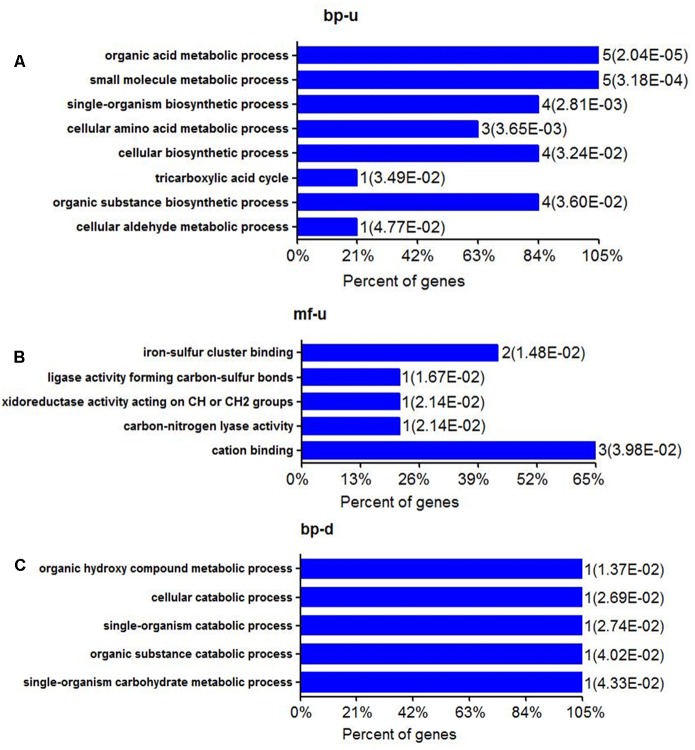
Biological processes and molecular function of differential expressed proteins of *E. tarda* in alanine treatment at the protein level. **(A,B)** Gene ontology (GO) shows up-regulation of biological processes and molecular functions **(C)** GO of BP in down-regulated proteins. MF can’t be enriched.

The most up-regulation of biological processes includes 5 proteins (account for 23.8%) related to organic acid metabolic process and another 5 proteins (account for 23.8%) related to small molecule metabolic processes (**Figure 1A**). In addition, proteins related to single-organism biosynthetic process (4 proteins, accounting for 19%), cellular amino acid metabolic (3 proteins, accounting for 14%), cellular biosynthetic processes (4 proteins, accounting for 19%), and organic substance biosynthetic (4 proteins, accounting for 19%) were also up-regulated (**Figure 1A**). The molecular function of up-regulated proteins in response to alanine was enriched including iron-sulfur binding (2 proteins, accounting for 10.5%), cation binding (3 proteins, accounting for 15.8%) and 1 ligase activity forming carbon-sulfur bonds, oxidoreductase activity acting on -CH or -CH2 groups and carbon lyase activity, respectively (**Figure 1B**). We have noted that only one protein was enriched in several biological processes during the annotation, including organic hydroxyl compound metabolic process, cellular catabolic, and single- organism catabolic, organic substance catabolic and single-organism carbohydrate metabolic processes, respectively (**Figure 1C**). The down-regulation of molecular functioning cannot be enriched, possibly because few down-regulation proteins were quantitated in this study.

### KEGG Pathway Analysis of Differential Proteins upon Exogenous Alanine Administration

**FIGURE 2:**
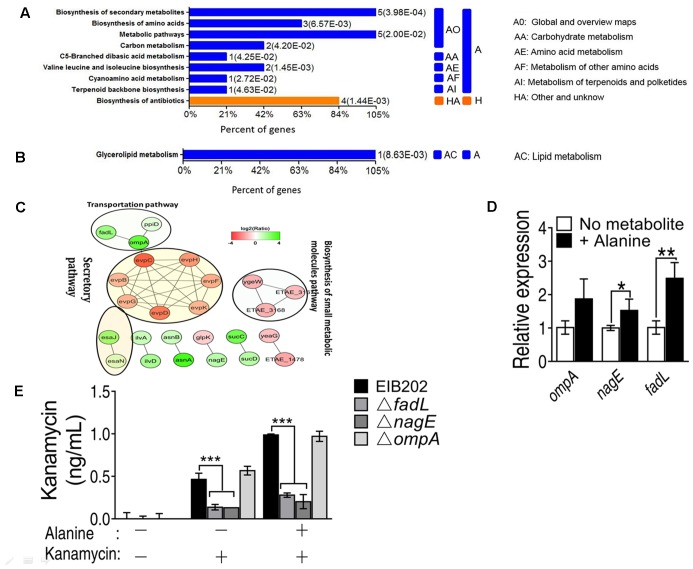
KEGG pathway enrichment of metabolic pathway shows **(A)** up and **(B)** down regulated proteins in the presence of exogenous alanine; **(C)** STRING software prediction of the protein–protein interaction pathways induced by alanine (including the secretory pathway, transportation pathway and biosynthesis of small metabolic molecules pathway); **(D)** QRT-PCR validation of the expression of three outer membrane proteins, OmpA, NagE and FadL; **(E)** Quantification of intracellular concentration of kanamycin in EIB202, or *fadL*, *nagE* and *ompA* single deletion mutants. ^∗^*p* < 0.05, ^∗∗^*p* < 0.01, and ^∗∗∗^*p* < 0.001 as determined by Student’s *t*-test.

KEGG metabolic pathway enrichment analysis made the networks with a *p*-value < 0.05 in the presence of alanine. KEGG pathway analysis was used to recognize the altered regulation of varied genes related to essential energy formation processes. The data of KEGG pathway are categorized into two broad categories according to OmicsBean analysis in up-regulated proteins, namely metabolism (A) and other unknown proteins (H) and shown in (**Figure 2A**). The metabolism category was subdivided into five categories (AO, AA, AE, AF, and AI) according to their functions. For example, five proteins involved in the biosynthesis of secondary metabolism and metabolic pathways, respectively; 2 proteins related to carbon metabolism, and 3 proteins associated with biosynthesis of amino acids. On the other hand, only one down-regulation protein was enriched in glycerolipid metabolism (**Figure 2B**).

The protein–protein interaction network plays fundamental biological pathways. Complex and multifunctional protein-protein networks were probed out via STRING software in this study. Resultantly we observed altered abundance of proteins associated with the number of biological processes as well actively involved into the constructions of protein–protein networks as shown in **Figure 2C**. The expression level of outer membrane proteins OmpA, FadL and NagE was increased. In contrast, the secretory pathway related proteins, such as EvpC, EvpB, EvpD, EvpG, EvpH, EvpF, and EvpK were down-regulated (**Figure 2C**).

To validate our proteomic results, the transcription of the *fadL* and *nagE* genes was increased in the presence of alanine except for the *ompA* gene (**Figure 2D**). In addition, the *nagE* and *fadL* knockout mutants had reduced intracellular kanamycin levels even in the presence of alanine in contrast to the *ompA* mutant. This indicates that *nagE* and *fadL* might be essential for the uptake of kanamycin by EIB202 (**Figure 2E**).

### Integrated Pathway Analysis of Alanine-Induced Proteome and Metabolome Change

**FIGURE 3:**
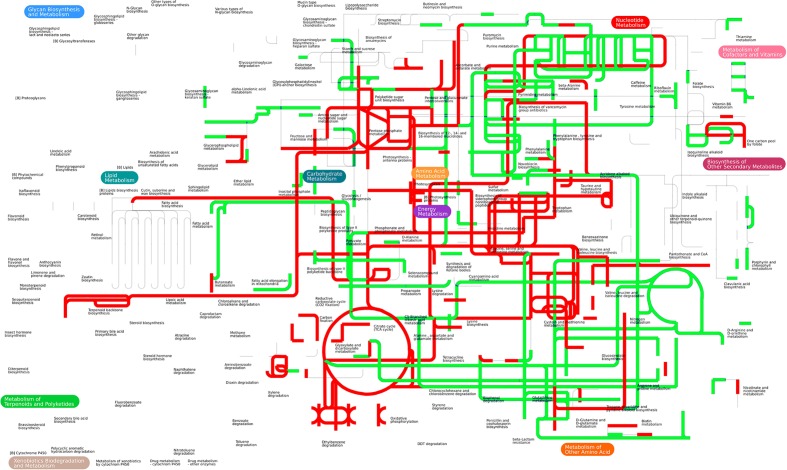
Integrated proteomic and metabolomics analysis in iPath. Forty differentially expressed proteins and 47 differentially expressed metabolites were simultaneously analyzed in iPath software. The color indicates expression change (red indicates up-regulation, and green indicates down-regulation).

The proteomic analysis uncovered several pathways that have been up-regulated by the exogenous alanine. We conducted an in-depth analysis by integrating altered abundance of metabolites (47 metabolites from our previous study) (Peng et al., 2015b) (**Supplementary Table 2**) and the altered abundance of proteins (40 proteins) (**Tables 1**, **2**) (**Supplementary Table 1**) in iPath, a versatile software for visualizing integrated pathways in metabolites and proteins (Letunic et al., 2008). Interestingly, pathways enriched from proteomic data is well consistent with pathways enriched from metabolomic data, e.g., the up-regulation of TCA cycle, carbon metabolism and amino acid metabolism. Of note, the integrated pathway analysis also enriched the riboflavin metabolism, which is unexpected (**Figure 3**).

Riboflavin (vitamin B_2_) is the universal precursor of flavin mononucleotide (FMN) and flavin adenine dinucleotide (FAD), the coenzymes of flavorproteins essential for many metabolic pathways including fatty acid metabolism, TCA cycle and electron transport chain (Massey, 2000; Lin et al., 2014). The autoxidation of flavorprotein through FADH_2_ oxidation is responsible for the production of both O_2_^-^ and H_2_O_2_, the important source of ROS generation (Imlay, 2013). Therefore, the connection of exogenous alanine to riboflavin metabolism implied that alanine might have additional roles in facilitating antibiotic killing of multidrug-resistant bacteria.

### Role of ROS in Synergistic Killing of Kanamycin with Alanine

**FIGURE 4:**
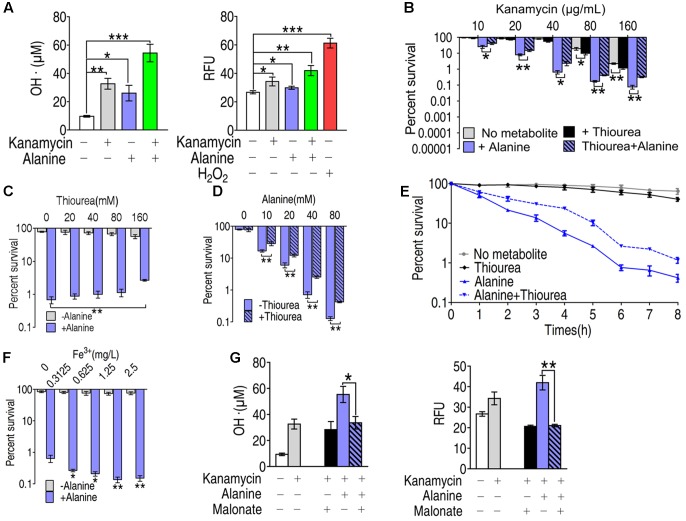
Role of ROS in the synergistic killing of kanamycin with alanine. **(A)** Hydroxyl radical was quantified in EIB202 in the presence of kanamycin, alanine and/or glucose as indicated by colorimetric method (left panel) and by fluorescence (right panel). **(B)** Percent survival of EIB202 in the presence of thiourea (160 mM), alanine (40 mM) or both. **(C)** Percent survival of EIB202 in the presence or absence of thiourea (160 mM) and the indicated concentration of alanine. **(D)** Percent survival of EIB202 in the presence or absence of alanine and the indicated concentration of thiourea (160 mM). **(E)** Percent survival of EIB202 in the presence or absence of kanamycin (40 μg/mL), thiourea (160 mM) and alanine (40 mM) at the indicated time point. **(F)** Percent survival of EIB202 in the presence or absence of alanine and the indicated concentration of Fe^3+^(C_6_H_10_FeNO_8_). **(G)** Effect of malonate (20 mM) on hydroxyl radical concentration in the presence or absence of alanine and/or kanamycin by two different assays as by colorimetric method (left panel) and fluorescence (right panel). ^∗^*p* < 0.05, ^∗∗^*p* < 0.01, and ^∗∗∗^*p* < 0.001 as determined by Student’s *t*-test.

To investigate the role of ROS in alanine-enabled killing of multidrug-resistant bacteria by kanamycin, we quantified the free radicals by colorimetric method (**Figure 4A**, left panel) and by fluorescence (**Figure 4A**, right panel) in EIB202 treated with alanine, kanamycin or alanine plus kanamycin. Kanamycin or alanine alone increased the production of free radicals by two different ROS quantification assays. The combination of kanamycin and alanine dramatically increased the ROS production determined by both of the assays (**Figure 4A**). Consistent with our previous results (Peng et al., 2015b), alanine plus kanamycin greatly increased the killing efficacy of kanamycin (**Figure 4B**). But this synergistic effect is partly abolished by thiourea in a dose dependent manner, and thiourea weakly promoted kanamycin-mediated killing (**Figure 4C**). The function of thiourea in protecting bacteria from ROS-dependent death was also concentration- and time- dependent (**Figures 4D,E**). In addition, exogenous iron, which dramatically increased ROS production, enhanced the alanine-enabled killing by kanamycin, indicating the role of ROS in metabolite-enabled killing of multidrug-resistant bacteria (**Figure 4F**). Moreover, malonate, a competitive inhibitor for succinate dehydrogenase, largely reduced the ROS production, indicating the essentiality of TCA cycle in alanine plus kanamycin-mediated killing, and the possible role of TCA cycle in alanine-triggered ROS production (**Figure 4G**).

### ROS Generation in the Presence of Alanine

**FIGURE 5:**
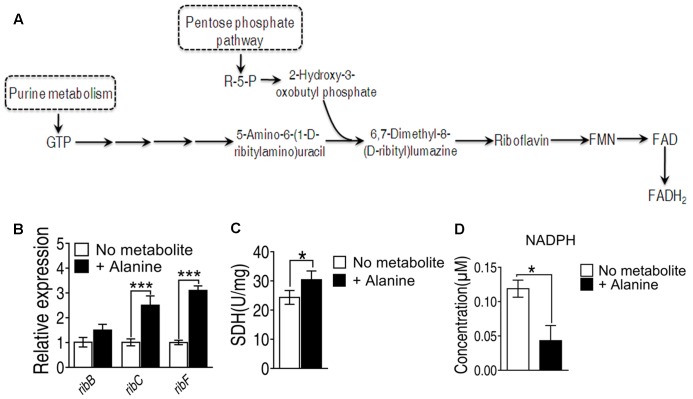
**(A)** Metabolic pathway of riboflavin metabolism and genes involved in each reaction. **(B)** Transcriptional level of genes of riboflavin metabolism; **(C)** Succinate dehydrogenase activity was measured in the presence of alanine. **(D)** Intracellular concentration of NADPH induced by alanine. ^∗^*p* < 0.05, ^∗∗^*p* < 0.01, and ^∗∗∗^*p* < 0.001 as determined by Student’s *t*-test.

Since the genes involved in riboflavin metabolism are essential genes, we didn’t adopt gene deletion studies in the present study. To investigate alanine-triggered ROS production is through riboflavin metabolism, we measured the transcriptional activity of the genes in this pathway including *ribB, ribC* and *ribF*, whose functions are to convert 6,7-dimenthyl-8-(D-ribityl)lumazine to riboflavin, to convert riboflavin to FMN, and to convert FMN to FAD, respectively (**Figure 5A**). The transcriptional level of these three genes are increased at least two folds in the presence of alanine (**Figure 5B**). In addition, FADH_2_ is also generated in the TCA cycle during the conversion of succinate to fumarate by succinate dehydrogenase (SDH). Exogenous alanine increased enzymatic activity of SDH for more than 20% (**Figure 5C**). Meanwhile, the intracellular concentration of NADPH, providing the reducing equivalents for biosynthetic reactions and the oxidation-reduction involved in protecting against the toxicity of ROS, was decreased by alanine (**Figure 5D**) (Rush et al., 1985). These data implied that alanine likely promote ROS production through FADH_2_ oxidation, and inhibition of ROS degradation.

### Exogenous Alanine Represses Antioxidants Defense System

**FIGURE 6:**
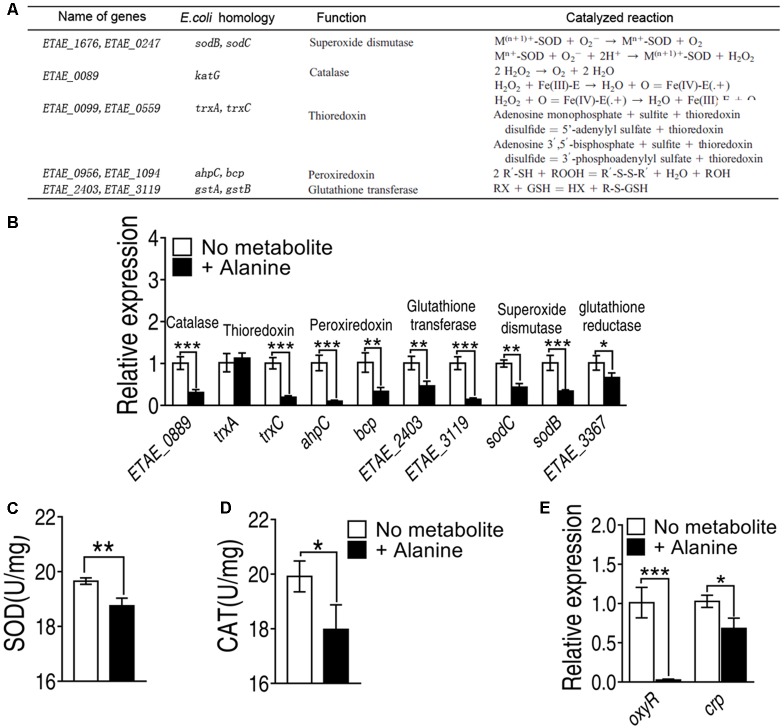
Alanine represses genes with antioxidant functions **(A)** Genes with antioxidant functions present in *E. tarda*, their homologues in *Escherichia coli*, their functions and the catalyzed reactions of the encoded enzymes. **(B)** Transcriptional level of genes of major classes of antioxidants. **(C)** SOD activity and **(D)** catalase activity in the presence of exogenous alanine (40 mM). **(E)** Transcriptional level of *oxyR* and *crp* in the presence of alanine. ^∗^*p* < 0.05, ^∗∗^*p* < 0.01, and ^∗∗∗^*p* < 0.001 as determined by Student’s *t*-test.

The elevated ROS may be resulted from the failure of antioxidants, molecules inhibit oxidation of other molecules, is inhibited. To investigate whether antioxidants are repressed by exogenous alanine, we measured the transcriptional level of the five major antioxidants defense system inside the cell, including superoxide dismutase, catalase, thioredoxin, peroxiredoxin and glutathione transferase, where glutathione peroxidase was not present in *E. tarda* (**Figure 6A**). Exogenous alanine significantly decreased transcription of *ETAE_0889*, *trxC*, *ahpC*, *bcp*, *ETAE_2403*, *ETAE_3119*, *sodC*, and *sodB* for at least twofolds (**Figure 6B**). Furthermore, the enzymatic activity of SOD was decreased by 20%, and catalase activity was decreased 52% in the presence of exogenous alanine (**Figures 6C,D**). Although the regulation of antioxidant enzymes in *E. tarda* is not well understood, we searched for their homologous genes in *Escherichia coli* (**Figure 6A**). *ETAE_0889, ahpC, trxC and ETAE_3367* were under the control of *oxyR*, whose transcriptional level was significantly repressed in the presence of alanine (**Figure 6E**). In addition, *oxyR* was positively regulated by CRP-cAMP that was only slighted decreased in response to alanine (**Figure 6E**). These data suggested that alanine inhibits the enzymes that degrade ROS through down-regulating the transcription of *oxyR*.

### Exogenous Alanine Connects Riboflavin Metabolism via TCA Cycle, Glucogenesis Pathway, and Pentose Phosphate Metabolism

**FIGURE 7:**
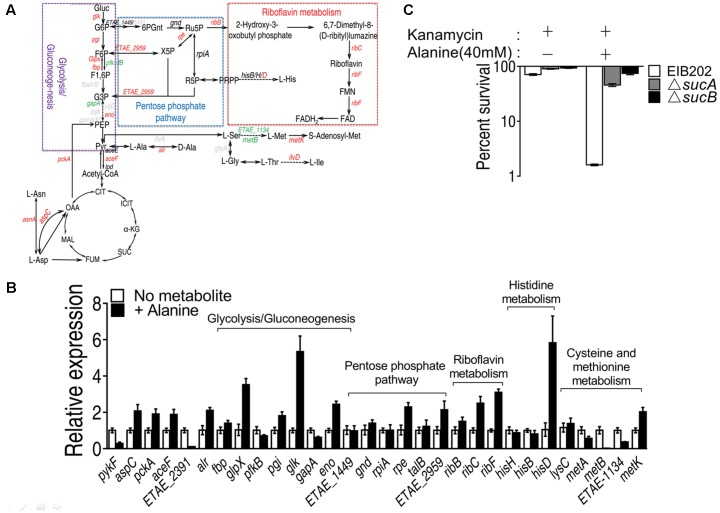
Alanine metabolism connected to riboflavin metabolism through TCA cycle, glucogenesis pathway and pentose phosphate pathway. **(A)** Metabolic network in association with exogenous alanine; **(B)** Transcriptional level of genes of alanine, aspartate and glutamate metabolism, TCA cycle, glycolysis/glucogenesis pathway, pentose phosphate pathway, histine metabolism and cysteine methionine metabolism; **(C)** Percent survival of EIB202, Δ*sucA* or Δ*sucB* in the presence of kanamycin or alanine plus kanamycin. ^∗^*p* < 0.05, ^∗∗^*p* < 0.01, and ^∗∗∗^*p* < 0.001 as determined by Student’s *t*-test.

Alanine metabolism could be connected to riboflavin metabolism through TCA cycle, glucogenesis pathway and pentose phosphate metabolism (**Figure 7A**). By QRT-PCR, we have confirmed the increase in alanine, aspartate and glutamate metabolism and TCA cycle, being consistent with our previous results (**Figure 7B**). The interruption of TCA cycle by knocking out *sucA* or *sucB* abolished the killing by kanamycin even in the presence of alanine (**Figure 7C**) (Peng et al., 2015b). In addition, the expression level of genes in pentose phosphate pathway, including *rpe* and *ETAE_2959*, was significantly increased, and the genes of glycolysis/glucogenogenesis pathway, including *glpX, pgi, glk*, and *eno*, were also increased. Since the expression of genes, including *ETAE_1848* and *gnd*, the product of which metabolizes D-glucose-6-phophate to D-ribulose-5-phosphate remain unaffected, the metabolism likely flows from Fru-6-phosphate or D-fructose 1,6-phosphate to D-ribulose-5-phosphate, which then enters the riboflavin metabolism by *ribB* (**Figure 7B**). These results implied that the exogenous alanine promote the metabolic flow of alanine to TCA cycle, followed by glucogenesis pathway, and then to pentose phosphate pathway, and riboflavin metabolism and histidine metabolism.

## Discussion

Modulating metabolism to increase the efficacy of antibiotics to kill multidrug-resistant bacteria is an attractive strategy to tackle the superbugs (Allison et al., 2011; Peng et al., 2015a,b; Su et al., 2015; Meylan et al., 2017), Exogenous alanine, glucose or fructose would increase the uptake of antibiotics to kill the multidrug-resistant bacteria, which nevertheless relied on the activation of TCA cycle (Allison et al., 2011; Peng et al., 2015a; Su et al., 2015). However, the mechanism underlying this strategy is not fully understood. To search for other pathways that may be behind this killing, we adopted proteomic approach to systematically investigate the proteome change in the presence of exogenous alanine. This leads to the novel finding that ROS contributes to alanine-enabled killing of antibiotic-resistant pathogens by aminoglycoside antibiotics, which is helpful not only to understand the high efficacy of kanamycin potentiated by exogenous alanine, but also to explore the synergistic effect of amino acids with other drugs including antibiotics.

**FIGURE 8:**
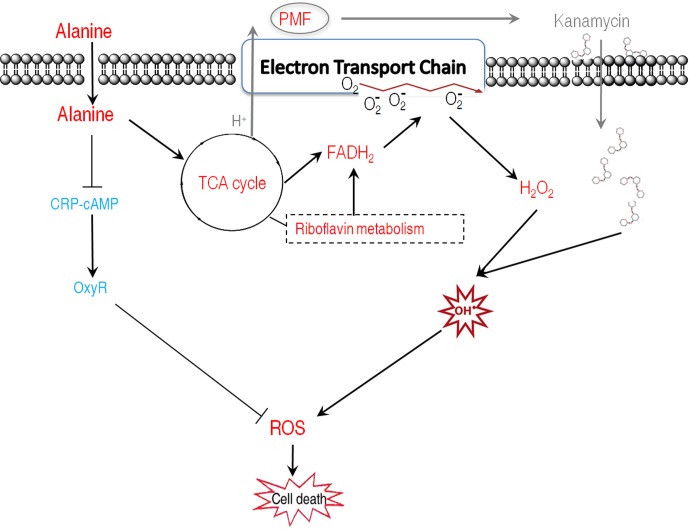
Proposed model for the dual roles of alanine in facilitating kanamycin to kill antibiotic-resistant bacteria. Exogenous alanine facilitates kanamycin to kill antibiotic-resistant bacteria through increased kanamycin uptake, and enhanced ROS production. Meanwhile, alanine decreased the transcription of *oxyR*, which plays essential role in positively regulating antioxidant enzymes. Thus, the enhanced ROS production was achieved through FADH_2_ oxidation and repression of antioxidant function genes via OxyR in an unidentified manner. Red represents increase and blue represents decrease.

Our results showed that alanine metabolism promoted ROS generation through riboflavin metabolism using integrated analysis of proteomic and metabolomic data. The result of alanine in promoting ROS generation is unexpected since there is no study showing amino acids could trigger the generation of ROS. To demonstrate the possibility, alanine-related ROS action, ROS generation-related and degradation-related genes, and metabolic pathways were examined in the presence of exogenous alanine. These explorations led to the following findings: (1) The killing efficacy increase of kanamycin by alanine is, at least partially, due to the ROS synergistically produced; (2) Alanine promotes ROS production through FADH_2_ oxidation of riboflavin metabolism, and inhibits ROS degradation through the inhibition of antioxidants; (3) Exogenous alanine connects riboflavin metabolism via TCA cycle, glucogenesis pathway, and pentose phosphate metabolism. These findings not only present a novel mechanism that alanine increase the efficacy of kanamycin in killing antibiotic-resistant bacteria, but also unveil a new role of alanine, especially the synergistic effect of ROS with kanamycin. Whether antibiotics’ killing depend on ROS is still under debate due to the assay specificity to quantify intracellular ROS (Kohanski et al., 2007; Keren et al., 2013; Liu and Imlay, 2013; Dwyer et al., 2015; Van Acker and Coenye, 2017), where the assay to quantify intracellular ROS was challenged. Redox-sensitive GFP (roGFP) allows dynamic measurements of intracellular redox potential changes under oxidative stress in bacteria. Using the roGFP biosensor no redox potential changes due to ROS production were found under antibiotics treatment in Gram-negative bacteria (van der Heijden et al., 2016). This roGFP biosensor was later engineered to monitor bacillithiol redox potential changes in the Gram-positive pathogen *Staphylococcus aureus*, but did not show redox changes in response to antibiotics (Loi et al., 2017). In the present study, by two different ROS quantification assays, we found that kanamycin, an aminoglycoside, increase the production of hydroxyl radicals, which was exaggerated in the presence of alanine. Interestingly, thiourea attenuated the kanamycin-mediated killing exaggerated by the synergy of alanine and kanamycin, but not the killing caused only by kanamycin. Instead, it weakly promotes the killing (**Figure 4C**). Thus, together with our previous finding (Peng et al., 2015a), we would like to propose a model for the kanamycin/alanine-mediated killing of antibiotic-resistant pathogens through generation of ROS by kanamycin: exogenous alanine promotes the TCA cycle and the generation of NADH as well as PMF. The enhanced TCA cycle promotes glucogeogenesis, pentose phosphate metabolism and riboflavin metabolism, and thus increases FADH_2_ generation. Meanwhile, antioxidants are inhibited. Increased FADH_2_ triggered the generation of more ROS. Together with the fact that the expression of antioxidants is inhibited, intracellular ROS get elevated, resulting in cell death (**Figure 8**). Interestingly, alanine alone has no effects on bacterial growth (data not shown), indicating that the amount of ROS promoted by alanine was not lethal or kept at a certain level that has no damage to the cells. Similarly, the ROS promoted by kanamycin didn’t display the killing efficacy as shown in **Figure 4C**, where thiourea inhibited alanine-triggered potentiation instead slightly promoted the kanamycine-mediated killing. Possible explanations include that ROS-mediated killing is related to not only its concentration, but also how ROS was generated.

Another interesting finding is that the alanine treatment did decrease the expression of several antioxidant enzymes, and their master regulators *oxyR*. Since *oxyR* is positively regulated by CRP-cAMP, it is highly possible that alanine may exert inhibitor effects on CRP-cAMP. Our data suggested that alanine has minor effect on the transcription of CRP. Thus, alanine could negatively regulate cAMP level. cAMP is inversely correlated with intracellular ATP level in the presence of glucose (Stulke and Hillen, 1999). But whether alanine has similar mode of action waits for further investigation.

In addition, the proteomics approach identified three elevated outer membrane proteins, OmpA, FadL and NagE in the presence of exogenous alanine. In our previous report, we showed that alanine promotes the TCA cycle through increased PMF, which positively regulates channels to open for aminoglycosides. However, the protein that is responsible for the increased uptake of aminoglycosides is still yet to be identified. The three proteins are conserved among Gram-negative bacteria. Whereas their contributions to the antibiotic resistance is not known in *E. tarda*, they have been well characterized in *Escherichia coli*. OmpA is a porin and represents an important molecule in bacterial pathogenesis (Hu et al., 2015). Decrease of OmpA is related to elevated minimum inhibitory concentration (Liu et al., 2010; Martınez-Puchol et al., 2015), and was reported in kanamycin-resistant bacteria (Zhang et al., 2015). FadL is long-chain fatty acid transport protein, whose expression is tightly associated with antibiotic resistance, being revealed in several studies. The expression level of FadL was down-regulated in kanamycin-, streptomycin- and nalidixic acid-resistant bacteria (Smith and Romesberg, 2007; Li et al., 2008, 2015; Lin et al., 2008, 2010). The *nagE* gene encodes the *N*-acetylglucosamine (GlcNAc)-specific transporter of the phosphotransferase system, producing intracellular GlcNAc-6-P. *nagE* mutant is resistant under all the conditions tested to even the highest doses of streptozotocin (Lengeler, 1980). *N*-acetyl-D-glucosamine induces the expression of multidrug exporter genes, *mdtEF* (Hirakawa et al., 2006), suggesting that the elevated NagE increases sensitivity to antibiotics through inhibition of *mdtEF* expression. Taken together, the elevation of three outer membrane proteins is associated with increased susceptibility to antibiotics. And in our current study, we confirmed that the two of the OMPs, *nagE* and *fadL* may mediate the transportation of kanamycin through the membrane. But whether these two proteins directly involved requires further interaction analysis of the proteins and antibiotics.

The synergistic effects of alanine and kanamycin in killing antibiotic-resistant bacteria with high efficiency granted potential use in clinic. Alanine, as a non-essential amino acid, was widely used as nutrient supplementation to patients with hypoglycaemia (Storgaard et al., 2016). Supplementation of 40 grams of L-alanine and 10 grams of glucose with NPH insulin before bed maintained healthy blood glucose levels more consistently than having a snack with the NPH to patients with type 1 diabetes (Desjardins et al., 2014). However, the report for the use of alanine in treating microbial infection is rare. Our previous report (Peng et al., 2015b) have demonstrated the use of the alanine and kanamycin combinations to treat mice infection by multidrug resistant bacteria, which was effective. The dose we used was 3 g/kg, which is higher than the dose used for patients with hypoglycaemia. We did not observe any abnormal symptoms for mice administrated at this dose of alanine alone (unpublished data). But we need to perform further experiment like pharmcokinetics and pharmacodynamics of alanine, kanamycin or alanine plus kanamycin to clear demonstrate their metabolism *in vivo* before the actual application in clinic.

In summary, the present study demonstrates that alanine has additional two new roles in facilitating kanamycin killing of antibiotic-resistant bacteria: synergistically generating ROS with kanamycin, and increasing membrane permeability through the regulation of outer membrane proteins. Thus, our study advances the previous studies in elucidating the mechanism of metabolite-enabled killing of antibiotic-resistant bacteria.

## Materials and Methods

### Bacterial Strains and Culture Conditions

*Edwardsiella tarda* strain, EIB202, used in this study was obtained from professor Yuanxin Zhang, East China University of Science (Wang et al., 2009). EIB202 were grown at 30°C for 24 h in 50 mL LB broth in 250 mL flasks. Bacterial cells were collected by centrifugation at 8,000 rpm for 5 min. The samples were then washed with sterile saline three times and suspended in M9 minimal media containing 10 mM acetate, 1 mM MgSO_4_ and 100 μM CaCl_2_, diluted to OD600 of 0.2. Alanine with 40 mM was added or not added as test and control groups, respectively, and then incubated at 30°C for 6 h, 200 rpm. The cultures were collected for analysis of liquid chromatography tandem-mass spectrometry (LC-MS) and QRT-PCR.

### Protein Extraction, Trypsin Digestion and iTRAQ Labeling

Total cellular proteins were extracted as previously described (Li et al., 2016). In brief, the collected cells were washed with PBS twice. After resuspension in diluted 10-fold SDT buffer (4% SDS, 0.1 M DTT and 0.5 MTEAB), the cells were sonicated for 10 min using an ultrasonic bath (Shumei Inc., Kunshan, China) and boiled for 10 min. Supernatants were separated by centrifuging at 12,000 *g* for 30 min at 4°C. The concentration of proteins in the supernatants were measured by the use of BCA assay (Thermo Fisher Scientific) and then stored at -20°C for further proteomic analysis. About 100 μg proteins of each group were dissolved in 8 M urea, 0.5 M TEAB buffer, and be reduced in 5mM Tris (2-carboxyethyl) phosphine (TCEP) at 37 °C for 1 h and then alkylated with 25 mM iodoacetamide (IAA) at room temperature in the dark for 45 min. The reduced protein samples were diluted to less than 1 M urea by triethylammonium bicarbonate (TEAB) buffer and digested to peptides by sequencing grade modified trypsin (Promega, United States) at a 1:50 ratio at 37°C overnight. The digested peptides were labeled and pooled together using iTRAQ kits as manufacture’s instruction and then desalted by Strata X C18 SPE column (Phenomenex, Aschaffenburg, Germany).

### LC-MS/MS

The desalted peptides sample was firstly separated to six fractions by Thermo DINOEX Ultimate 3000 BioRS with Durashell C18 column (5 μm, 100 Å, 4.6 × 250 mm) and then submitted to analysis by AB SCIEX nano LC-MS/MS (Triple TOF 5600 plus, AB SCIEX, Concord, ON, United States) with an Eksigent nanoLC-Ultra 2D System combined with the cHiPLC-nanoflex system as previously described setting (Lin et al., 2015). The raw data were interpreted by Proteinpilot 5.0 version against the *Edwardasiella tarda* database with following search parameters: iodoacetamide cysteine alkylation, trypsin digestion, iTRAQ peptide labeled, detected protein threshold > 0.05 and the false discovery rate (FDR) < 0.05. The identified proteins with at least two peptides matched were considered for further analysis. For quantification, the iTRAQ average reporter ion ratio ≥ 1.5 (increase) or ≤0.668 (decrease) and both *p*-value < 0.05 between two biological repeats were deemed to have a significant change.

### Bioinformatics Analysis

The altered abundance of proteins was further analyzed with bioinformatics methods such as GO (Gene ontology) functional annotation, KEGG pathways and protein–protein interaction network prediction as previously described. Briefly, GO annotation and KEGG pathway were analyzed with the online software OmicsBean^1^ (Li et al., 2017). The protein–protein interaction prediction network was analyzed by online software STRING v10^2^ and revised using Cytoscape 3.5.0 version^3^ (Shannon et al., 2003; Szklarczyk et al., 2015).

### QRT-PCR

The bacterial cells were harvested. The total RNA of each sample was isolated with Trizol (Invitrogen, United States). Reverse transcription-PCR was carried out on a PrimeScript^TM^ RT reagent Kit with gDNA eraser (Takara, Japan) with 1 mg of total RNA according to manufacturer’s instructions. QRT-PCR was performed in 384-well plates and each well contained a total volume of 10 mL liquid including 5 mL 2 × SYBR Premix Ex Taq^TM^, 2.6 μL PCR-grade water, 2 μL cDNA template and 0.2 μL each pair of primers (10 mM). The primers are listed in **Supplementary Table 3**. All the samples were performed on LightCycle 480 system (Roche, Germany) according to the manufacturer’s instructions and four independent samples were assayed for both control-group and test-group. The cycling parameters were listed as follows: 95°C for 30 s to activate the polymerase; 40 cycles of 95°C for 10 s; 60°C for 30 s; Fluorescence measurements were performed at 70°C for 1 s during each cycle. Cycling was terminated at 95°C with a calefactive velocity of 5°C per second and a melting curve was obtained. To analyze the relative expression level of target gene, we converted the data to percentages relative to the value of no treatment group.

### Succinate Dehydrogenase (SDH) Enzymatic Activity Assay

A single bacterial colony was grown in 50 ml LB broth in 250 ml flasks for 24 h at 30°C. After centrifugation at 8,000 3 rpm for 5 min, samples were washed twice with 30 ml sterile saline and re-suspended in M9 minimal media supplemented with 10 mM acetate, 1 mM MgSO4, and 100 mM CaCl2 to 0.2 at OD600. Reaction samples were added to metabolites and antibiotic and incubated at 30°C for 6 h. After incubation, collected cells and re-suspended in sterile saline(OD600 = 1.0), 1 mL samples were collected, centrifugation at 8,000 3 rpm for 5 min, the pellet was collected. To measure the enzymatic activity of SDH, pellet was re-suspended in PBS and broke down by sonication for 2 min at a 200 W power setting on ice. The samples were centrifuged at 12,000 rpm for 10 min to remove insoluble material. Supernatant containing 400 μg total proteins were transferred to the SDH reaction mix (0.5 mM MTT, 13 mM PMS, 5 mM sodium succinate,50 mM PBS) to a final volume of 200 μL in 96-well plate. Subsequently, the plate was incubated at 37°C for 5 min for SDH assay, then either of the plate was measured at 566 nm for colorimetric readings. The plate was protected from light during the incubation.

### Quantification of ROS Production

Reactive oxygen species production was quantified by two different assays. For quantification of ROS by Hydroxyl Radical Colorimetric Assay Kit (GENMED, Shanghai), a single colony of EIB202 was inoculated in TSB medium, grown at 30°C for 24 h. The culture was collected by centrifugation, washed three times and re-suspended in minimal medium (M9 medium). The bacteria were incubated with chemicals as indicated for another 6 h at 30°C. After incubation, bacteria were collected, and was adjusted OD_600_ to 1.0. The bacteria were then lysed completely by sonication. After centrifugation, the supernatant collected for the following assay. Cell lysates were mixed with fluorescence solution, and then incubated at room temperature for 30 min, which was subsequently mixed with hydroxyl radical initiator followed by Fenton reagent. After shaking, the sample was immediately read in a fluorescent microplate reader with excitation at 480 nm and emission at 530 nm. The concentration was calculated from the standard curve, which was prepared with the standards.

For ROS quantification by fluorescence, cells were grown in 50 mL LB in 250 mL baffled flasks at 30°C and 200 rpm until OD600 = 0.2. Cells were collected and washed, then treated with alanine, kanamycin or both or inhibitors, where H_2_O_2_(5 mM) serves as positive control. After treatment, cells were washed twice with prewarmed PBS and then incubated with 10 μM carboxy-H2DCFDA (Sigma, United States) at 37°C for 30 min in the dark. The cells were then washed twice with PBS and analyzed by a microplate reader (Varioskan LUX, Thermo Scientific, United States) at an excitation and emission wavelength of 495 and 525 nm, respectively.

### Antibiotic Bactericidal Assays

Antibacterial assay was carried out as described previously (Peng et al., 2015b; Su et al., 2015). Bacterial cells were collected by centrifugation at 8,000 rpm for 5 min. The samples were then washed with sterile saline three times and suspended in M9 minimal media containing 10 mM acetate, 1 mM MgSO_4_ and 100 μM CaCl_2_, diluted to OD600 of 0.2. Forty mM alanine or/and 40 μg kanamycine were added, and incubated at 30°C for 6 h, 200 rpm. To determine bacterial counts, 100 μL of cultures were removed, and then serially diluted. Aliquot 10 μL of each dilution was plated in TSB agar plates and incubated at 30°C or 37°C for 18 – 22 h. The plates only with 20 – 200 colonies were counted and CFU/mL was calculated.

### Quantification of Intracellular NADPH Concentration by LC-MS/MS

A single bacterial colony was grown in 50 mL LB broth in 250 mL flasks for 24 h at 30°C. After centrifugation at 8,000 rpm for 5 min, samples were washed twice with 30 mL sterile saline and re-suspended in M9 minimal media supplemented with 10 mM acetate, 1 mM MgSO_4_, and 100 mM CaCl_2_ to 0.2 at OD600. Reaction samples were added to metabolites and antibiotic, and incubated at 30°C for 6 h. After incubation, cells were collected and re-suspended in sterile saline (OD600 = 1.0). 1mL samples were collected by centrifugation at 8,000 rpm for 5 min. The pellet was re-suspended in 1 mL 50% acetonitrile, and then disrupted by sonication for 12 min, followed by centrifugation at 12,000 rpm for 10 min at 4°C twice. The supernatant was collected and analyzed by UPLC-MS/MS.

Ultra performance liquid chromatography analysis was performed on a Waters ACQUITY UPLC system equipped with a CORTECS UPLC HILIC (100 mm × 2.1 mm, 1.6 μm; Waters Corp.). The sample was injected during the loading step by the loading pump and auto-sampler onto the column. Separation was using linear gradient elution with mobile phase A (100% acetonitrile) and B (5 mM ammonium acetate in ultrapure water, pH = 7) at a flow rate of 0.2 mL min-1. The gradient elution was as follows: 0–1 min, 95% A; 1–1.5 min, 40%A; 1.5–2.5 min, 40%A; 2.5–3 min, 95%A; 3–5 min, 95%A. The injection volume was 10 μL, and the column temperature was kept at 35°C. Mass spectrometry detection was carried out in QUATTRO PREMIER XE equipped with an electrospray ionization source operating in negative ionization mode (ESI-). The capillary voltage was set to 3,600 V; the cone voltage was set to 40 V. The extractor voltage and RF Lens were set at 1 and 0.1 V, respectively. The desolvation gas flow was set to 650 L h^-1^ at temperature of 450°C, the cone gas flow rate was set at 50 L h^-1^, and the source temperature was set at 130°C. Identification was measured in the multiple reaction monitoring mode (MRM), the precursor ion and quantifier ion of NADPH was 744 > 159.

## Author Contributions

BP conceived the research project. J-zY, Y-bS, X-mL, S-sL, W-xL, and FA performed the experiments. J-zY, Y-bS, X-mL, and JZ performed the data analysis. J-zY, Y-bS, JZ, and BP interpret the data and discussed the results. BP wrote the manuscript. All authors reviewed the manuscript.

## Conflict of Interest Statement

The authors declare that the research was conducted in the absence of any commercial or financial relationships that could be construed as a potential conflict of interest.

## Footnotes

1 http://www.omicsbean.com:88/

2 http://string-db.org/

3 http://cytoscape.org/

## Supplementary Material

TABLE S1 Proteomic data summary for EIB202 treated with or without alanine.

TABLE S2 Lists of altered metabolites with alanine treatment.

TABLE S3 Lists of QRT-PCR primers used in this study.
