# Supplementary material for: Alanine Enhances Aminoglycosides-Induced ROS Production as Revealed by Proteomic Analysis
Source: Front Microbiol. 2018 Jan 30;9:29. doi: 10.3389/fmicb.2018.00029 (PMC5797687; doi:10.3389/fmicb.2018.00029)
Supplement: TABLE S3 — Lists of QRT-PCR primers used in this study. [file Table_3.docx]

Table 3. Lists of QRT-PCR primers used in this study
